# Effect and safety of interventional recanalization in acute cerebral infarction with low NIHSS score due to anterior circulation large vessel occlusion and exploration of factors associated with futile recanalization

**DOI:** 10.3389/fneur.2025.1473306

**Published:** 2025-03-07

**Authors:** Wensheng Zhang, Weifang Xing, Yangchun Wen, Xiaojing Zhong, Jinzhao He

**Affiliations:** ^1^Heyuan People’s Hospital, Guangdong Provincial People's Hospital Heyuan Hospital, Heyuan, China; ^2^Heyuan Key Laboratory of Molecular Diagnosis and Disease Prevention and Treatment, Doctors Station of Guangdong Province, Heyuan People's Hospital, Heyuan, China; ^3^Department of Neurology, Guangdong Heyuan Traditional Chinese Medicine Hospital, Heyuan, China

**Keywords:** low NIHSS score, ischemic stroke, large vessel occlusion, interventional recanalization, futile recanalization

## Abstract

**Objective:**

To explore the efficacy and safety of successful interventional recanalization in patients with low NIHSS score acute cerebral infarction due to anterior circulation large vessel occlusion and influencing factors of futile recanalization.

**Methods:**

A retrospective analysis was conducted on the clinical data of patients with acute cerebral infarction due to anterior circulation large vessel occlusion treated in our hospital from January 2019 to December 2023. Statistical methods such as chi square test, t-test and non parametric test for statistical analysis were used.

**Results:**

A total of 445 patients were included in the study, including 32 in the low NIHSS score group and 413 in the non low NIHSS score group. There were statistical differences in NIHSS score at onset, preoperative ASPECT score, collateral circulation score, pathogenesis, effective recanalization rate, futile recanalization rate and 3-month postoperative mRS score between the two groups. There was no statistical difference in the incidence of complications such as symptomatic cerebral hemorrhage between the two groups. There were statistically significant differences in preoperative ASPECT score and collateral circulation score in terms of factors affecting futile recanalization in patients with low NIHSS score.

**Conclusion:**

Patients with acute cerebral infarction with anterior circulation large vessel occlusion and low NIHSS score had good therapeutic effect after successful interventional recanalization, and the safety was comparable to that of patients with non low NIHSS score. The factors that affecting futile recanalization in patients with low NIHSS score included preoperative ASPECT score and collateral circulation score.

## 1 Introduction

Acute cerebral infarction with large vessel occlusion is a disease with serious harm because it has high incidence rate, high disability rate and high mortality rate (1). In addition to intravenous thrombolysis, interventional recanalization is another most important and effective treatment method for acute cerebral infarction with anterior circulation large vessel occlusion (2). In patients with acute cerebral infarction with anterior circulation large vessel occlusion and National Institute of Health stroke scale (NIHSS) score ≥ 6 within 24 h of onset, when there is a salvageable ischemic penumbra that meets the intervention criteria of DAWN and DEFFUSE 3 studies, existing guidelines recommend interventional recanalization treatment for such patients (3–5). However, existing guidelines have not yet made advanced recommendations for interventional recanalization therapy in patients with acute cerebral infarction with anterior circulation large vessel occlusion whose NIHSS score is ≤5 within 24 h of onset (5, 6). There are still many unknowns regarding the efficacy, safety and influencing factors related to futile recanalization in patients with acute cerebral infarction and large vessel occlusion with low NIHSS score who received interventional recanalization. Our study aimed to explore the efficacy, safety and futile recanalization influencing factors of successful interventional recanalization in patients with low NIHSS score who suffered from acute cerebral infarction due to anterior circulation large vessel occlusion.

## 2 Materials and methods

### 2.1 Study population

**Figure 1:**
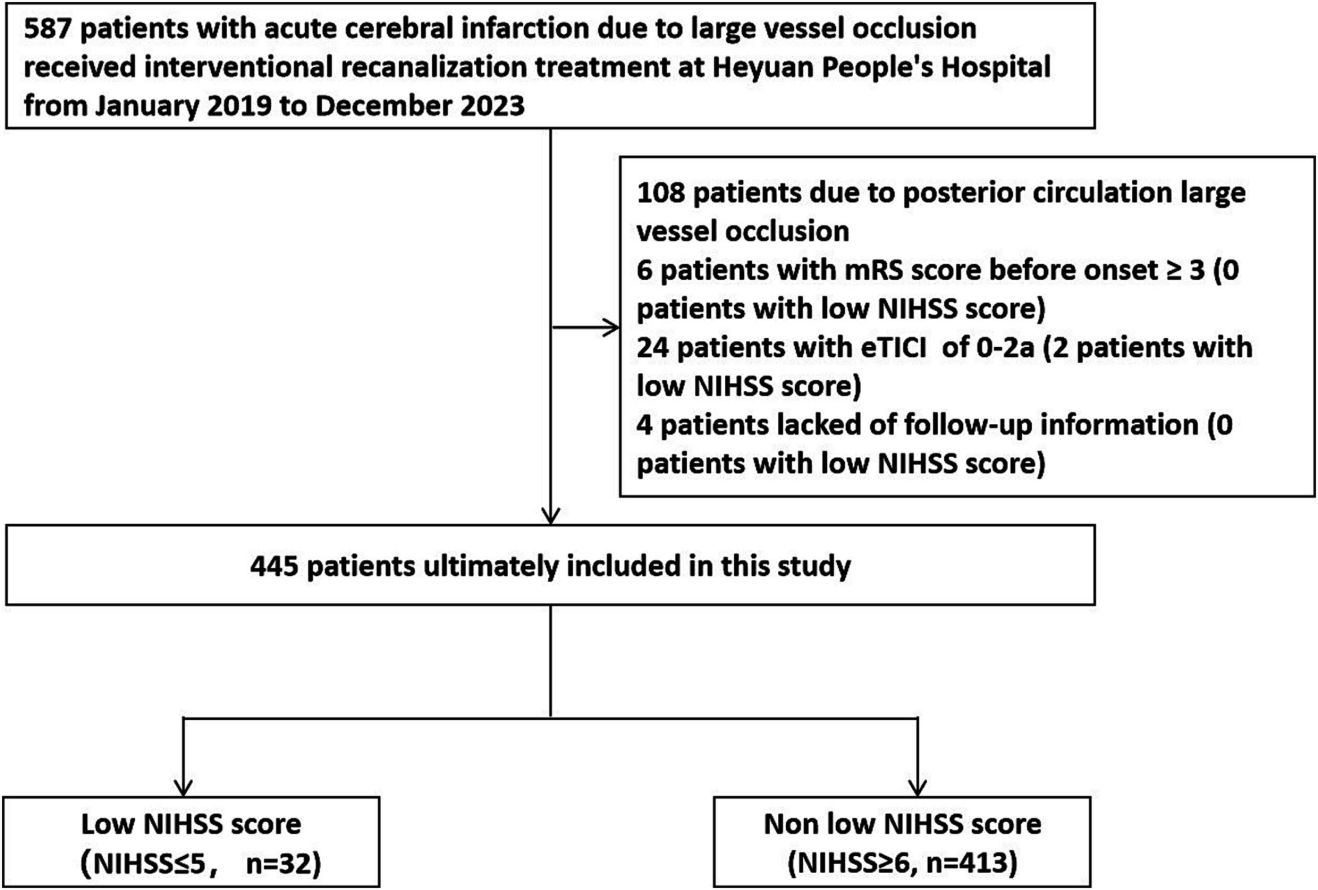
Research flowchart. mRS, modified Rankin Scale; NIHSS, National Institute of Health stroke scale.

We retrospectively analyzed 452 patients with acute cerebral infarction caused by anterior circulation large vessel occlusion who received successful interventional recanalization treatment at Heyuan People’s Hospital from January 2019 to December 2023. Inclusion criteria: (1) Age ≥ 18 years old; (2) NIHSS score at admission ≤5 points; (3) Preoperative Alberta Stroke Project Early Computed Tomography (ASPECT) score ≥ 6 points; (4) The time from onset to femoral artery puncture ≤24 h; (5) Cerebral angiography confirmed occlusion of large blood vessel in the anterior circulation; (6) The patient or family member signed and agreed to undergo interventional recanalization treatment; (7) Interventional recanalization was successful, and the extended thrombolysis in cerebral infarction (eTICI) grading of recanalized blood flow was 2b-3. Exclusion criteria: (1) Previous modified Rankin scale (mRS) score ≥ 3 points; (2) Interventional recanalization failed, with an eTICI grading of 0-2a for recanalized blood flow; (3) Patients or family members refused to undergo interventional recanalization treatment; (4) Instability of vital signs at onset of illness. The Ethics Committee of Heyuan People’s Hospital approved the acquisition of retrospective study patient data from the hospital’s clinical database for this study, and waived written informed consent. The research flowchart is shown in Figure 1.

### 2.2 Treatment methods

After imaging screening and evaluation, all patients were treated according to the existing guidelines of the American College of Cardiology and the Stroke Society. The first choice of anesthesia method was local anesthesia combined with sedation and analgesia. Anesthesia methods can be changed to general anesthesia if the condition required. After confirming occlusion of the anterior circulation large blood vessel by cerebral angiography examination, the interventional physicians selected one or two methods including stent thrombectomy or thrombus aspiration, based on the vascular pathway, location of the occluded vessel and possible pathogenesis. If necessary, balloon dilation or stent implantation can be used as remedy therapy. Postoperative management of patients was followed to existing guidelines.

### 2.3 Patient data collection

The demographic data and other relevant clinical data information of all enrolled patients were extracted from the hospital’s electronic database by uniformly trained researchers. Patients were followed up through telephone tracking, outpatient follow-up, inpatient follow-up and other methods.

### 2.4 Definition and classification of variables in data

A mRS score of 3–6 points 3 months after interventional recanalization was defined as futile recanalization, and an mRS score of 0–2 points was defined as effective recanalization. The site of vascular occlusion can be divided into 5 categories: M1 segment of the middle cerebral artery, M2 segment of the middle cerebral artery, internal carotid artery, anterior cerebral artery and anterior circulation tandem lesion. According to digital subtraction angiography (DSA) imaging, the American Society of Interventional and Therapeutic Neuroradiology/Society of Interventional Radiology (ASITN/SIR) scoring system was used for collateral circulation scoring. The degree of vascular recanalization was evaluated using the extended thrombolysis in cerebral infarction (eTICI) vascular recanalization grade. The eTICI grade of 2b-3 was defined as successful recanalization, while the eTICI grade of 0-2a was defined as failed recanalization. Symptomatic cerebral hemorrhage was defined as any type of cerebral hemorrhage, and the patient’s NIHSS score increased by ≥4 points from baseline or died. The pathogenesis can be divided into three types: large atherosclerosis, cardiogenic embolism and other causes.

### 2.5 Statistical processing

This study used SPSS 26.0 statistical software to conduct statistical analysis on the data. If the quantitative data conformed to a normal distribution, it was expressed as mean ± standard deviation (x ± s), and independent sample t-test was used for inter group comparison. Data that did not follow a normal distribution were described as median and interquartile intervals, and Wileoxon rank sum test was used for inter group comparison. Count data was expressed in percentage, and intergroup comparisons were conducted using chi square or Fisher’s exact test. A *p*-value less than 0.05 was considered statistically significant.

## 3 Results

### 3.1 Success rate of occluded vessel interventional recanalization in patients with low and non low NIHSS score

According to the research flowchart, the success rate of interventional recanalization in the low NIHSS score group was 94.12% (32/34), while the success rate of interventional recanalization in the non low NIHSS score group was 92.81% (413/445).

### 3.2 Clinical baseline data of two groups of patients with low and non low NIHSS score

**Figure 2:**
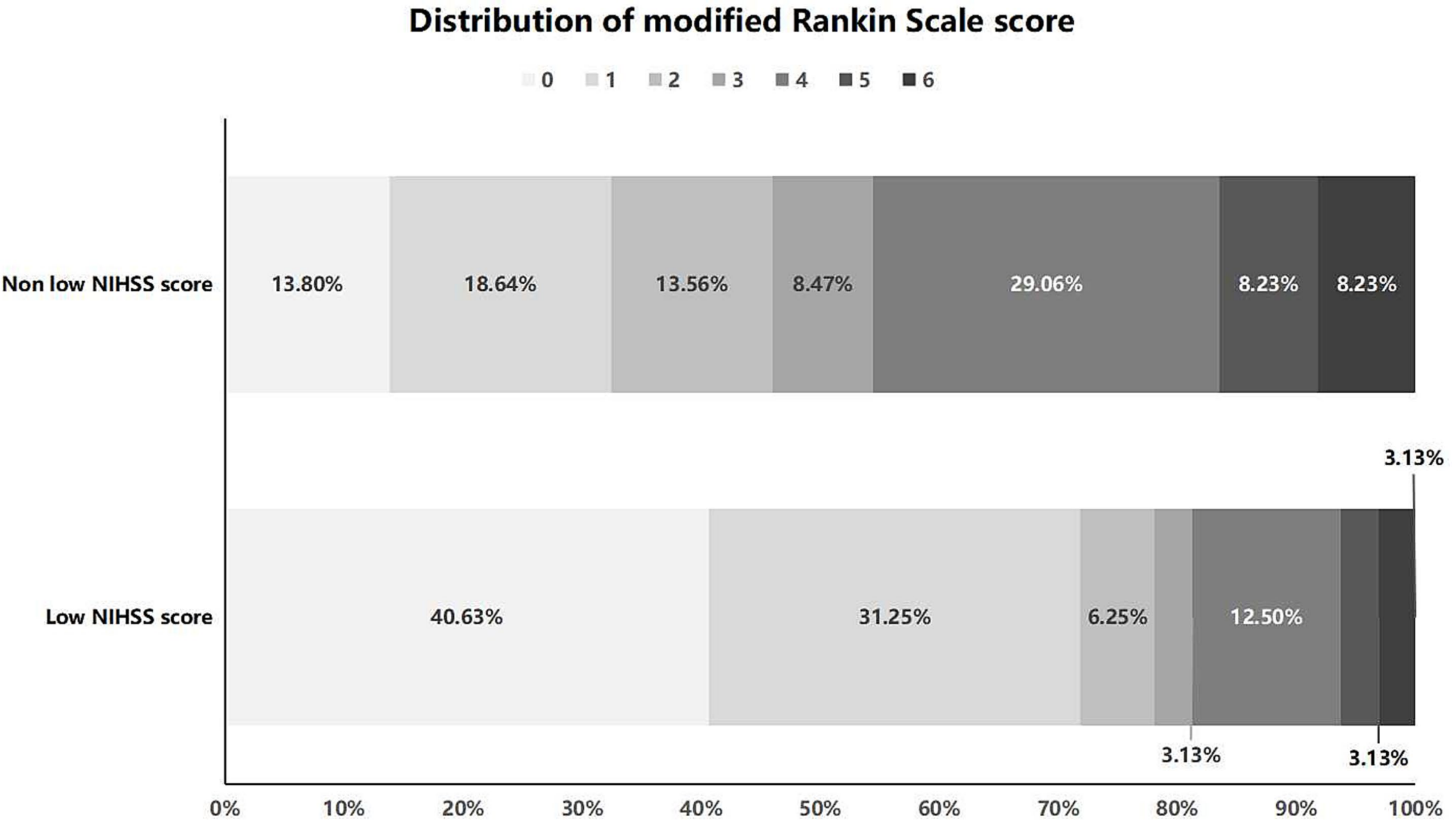
Distribution of mRS score at 3 months after interventional recanalization for patients in the low and non low NIHSS score group. NIHSS, National Institute of Health stroke scale.

A total of 445 patients were included, including 32 patients with low NIHSS score and 413 patients with non low NIHSS score. The average age of patients with low and non low NIHSS score was 63.44 ± 12.01 and 66.84 ± 11.60 years, respectively. There were statistically significant differences in NIHSS score, preoperative ASPECT score, collateral circulation score, pathogenesis, effective recanalization rate, futile recanalization rate and mRS score at 3 months after interventional recanalization between the two groups of patients (*p* < 0.05). The preoperative ASPECT score of patients in the low NIHSS score group was higher than that in the non low NIHSS score group. The overall collateral circulation score of patients in the low NIHSS score group was higher than that in the non low NIHSS score group. In terms of pathogenesis, patients in the low NIHSS score group had a higher proportion of large atherosclerosis and a lower proportion of cardiogenic embolism. The distribution of mRS score for two groups of patients 3 months after interventional recanalization is shown in Figure 2. There was no significant difference between two groups in age, sex, hypertension history, diabetes history, cerebral infarction history, coronary heart disease history, atrial fibrillation history, smoking history, drinking history, pre onset mRS score, receiving intravenous thrombolysis treatment, occluded vessel position, recanalization blood flow eTICI grading, time from onset to recanalization of occluded vessel and time from puncture to recanalization of occluded vessel (*p* > 0.05). The comparison of clinical baseline data between two groups of patients is shown in Table 1.

**Table 1:** Clinical baseline data of low and non low NIHSS score two groups of patients with acute cerebral infarction due to anterior circulation large vessel occlusion and received successful interventional recanalization.

Variables	Low NIHSS (*n* = 32)	Non low NIHSS (*n* = 413)	*p* value
Age, years, (mean ± standard deviation)	63.44 ± 12.01	66.84 ± 11.60	0.131
Gender, male, *n* (%)	25 (78.13)	278 (67.31)	0.206
Medical history			
Hypertension, *n* (%)	18 (56.25)	219 (53.03)	0.725
Diabetes, *n* (%)	4 (12.5)	77 (18.64)	0.386
Cerebral infarction, *n* (%)	6 (18.75)	67 (16.22)	0.710
Coronary heart disease, *n* (%)	1 (3.13)	53 (12.83)	0.105
Atrial fibrillation, *n* (%)	2 (6.25)	78 (18.89)	0.073
Smoking, *n* (%)	13 (40.63)	166 (40.19)	0.962
Drinking, *n* (%)	6 (18.75)	94 (22.76)	0.601
mRS score before onset, mean ± standard deviation	0	0.13 ± 0.49	0.109
The situation at the onset of stroke			
NIHSS score at onset, median (IQR)	4 (3–5)	12 (10–16)	<0.001
Preoperative ASPECT score, median (IQR)	9 (7–9)	7 (7–8)	0.001
Received intravenous thrombolysis treatment, *n* (%)	14 (43.75)	146 (35.35)	0.340
Location of occluded blood vessels, *n* (%)			0.782
M1 segment of Middle cerebral artery	17 (53.13)	202 (48.91)	
M2 segment of Middle cerebral artery	2 (6.25)	23 (5.57)	
Internal carotid artery	6 (18.75)	56 (13.56)	
Anterior cerebral artery	0 (0)	2 (0.48)	
Anterior circulation tandem lesion	7 (21.88)	130 (31.48)	
Collateral circulation score, median (IQR)	3 (3–3.75)	3 (2–3)	0.001
eTICI grading of recanalization blood flow, *n* (%)			0.885
2b	6 (18.75)	93 (22.52)	
2c	2 (6.25)	25 (6.05)	
3	24 (75)	295 (71.43)	
Time nodes			
Time from onset to recanalization of occluded blood vessel, min, median (IQR)	590 (336.5–875.75)	546 (411.5–760.5)	0.797
Time from puncture to recanalization of occluded blood vessel, min, median (IQR)	99 (70–150.25)	95 (70.5–127)	0.536
Pathogenesis			0.016
Large atherosclerotic	22 (68.75)	195 (47.22)	
Cardiogenic embolism	2 (6.25)	118 (28.57)	
Other reasons	8 (25.00)	100 (24.21)	

NIHSS, National Institute of Health stroke scale; mRS, modified Rankin Scale; ASPECT, Alberta Stroke Program Early CT Score; eTICI: extended thrombolysis in cerebral infarction.

### 3.3 Comparison of complications between two groups of patients with low and non low NIHSS score

There was no statistically significant difference in the incidence of symptomatic cerebral hemorrhage (3.13% vs. 7.02%), asymptomatic cerebral hemorrhage (9.38% vs. 12.35%), subarachnoid hemorrhage (9.38% vs. 4.6%), cerebral hernia (0 vs. 6.54%), stroke associated pneumonia (40.63% vs. 53.03%), deep vein thrombosis (0 vs. 3.63%) and gastrointestinal bleeding (12.50% vs. 12.59%) between the two groups of patients with low and non low NIHSS score (*p* > 0.05). The comparison of complications between the two groups of patients is shown in Table 2.

**Table 2:** Comparison of complications between low and non low NIHSS score two groups of patients with successful interventional recanalization in acute cerebral infarction with anterior circulation large vessel occlusion.

Variables	Low NIHSS (*n* = 32)	Non low NIHSS (*n* = 413)	*p* value
Symptomatic cerebral hemorrhage, *n* (%)	1 (3.13)	29 (7.02)	0.397
Asymptomatic cerebral hemorrhage, *n* (%)	3 (9.38)	51 (12.35)	0.620
Subarachnoid hemorrhage, n (%)	3 (9.38)	19 (4.60)	0.230
Brain Hernia, *n* (%)	0 (0)	27 (6.54)	0.136
Stroke associated pneumonia, n (%)	13 (40.63)	219 (53.03)	0.176
Deep vein thrombosis, *n* (%)	0 (0)	15 (3.63)	0.273
Gastrointestinal bleeding, *n* (%)	4 (12.50)	52 (12.59)	0.988

NIHSS, National Institute of Health stroke scale.

### 3.4 Comparison of prognosis between two groups of patients with low and non low NIHSS score

There was a statistically significant difference (*p* < 0.05) in effective recanalization, futile recanalization and mRS score at 3 months after interventional recanalization between the two groups of patients with low and non low NIHSS score. The effective recanalization rate and postoperative 3-month mRS score of the low NIHSS score group were higher than those of the non low NIHSS score group, while the futile recanalization rate was lower than that of the non low NIHSS score group. There was no statistically significant difference in all-cause mortality within 3 months after interventional recanalization between the two groups of patients (*p* > 0.05). The comparison of prognosis between the two groups of patients is shown in Table 3.

**Table 3:** Comparison of prognosis between low and non low NIHSS score two groups of patients with successful intervention recanalization in acute cerebral infarction with anterior circulation large vessel occlusion.

Variables	Low NIHSS (*n* = 32)	Non low NIHSS (*n* = 413)	*p* Value
Effective recanalization (mRS ≤ 2 points at 3 months after interventional recanalization), n (%)	25 (78.13)	190 (46.00)	<0.001
Futile recanalization (mRS ≥ 3 points at 3 months after interventional recanalization), n (%)	7 (21.87)	223 (54.00)	<0.001
mRS score 3 months after interventional recanalization, mean ± standard deviation	1.38 ± 1.72	2.78 ± 1.86	<0.001
Death due to all causes within 3 months after interventional recanalization, n (%)	1 (3.13)	34 (8.23)	0.301

NIHSS, National Institute of Health stroke scale.

### 3.5 Comparison of clinical baseline data between effective and futile recanalization groups in patients with low NIHSS score

According to the prognosis at 3 months after interventional recanalization, patients with low NIHSS score were divided into two groups: effective recanalization and futile recanalization. There was a statistical difference (*p* < 0.05) in the preoperative ASPECT score and collateral circulation score between the two groups of patients. The preoperative ASPECT score and collateral circulation score of the effective recanalization group were higher than that of futile recanalization group. There was no significant difference between the two groups in age, sex, hypertension history, diabetes history, cerebral infarction history, coronary heart disease history, atrial fibrillation history, smoking history, drinking history, pre onset mRS score, NIHSS score at onset, intravenous thrombolysis treatment, occluded vessel location, recanalization blood flow eTICI grading, time from onset to recanalization of occluded vessel, time from puncture to recanalization of occluded vessel and pathogenesis (*p* > 0.05). The comparison of clinical baseline data between effective and futile recanalization groups in patients with low NIHSS score is shown in Table 4.

**Table 4:** Clinical baseline data of two groups of patients with effective and futile recanalization who suffered from low NIHSS score acute cerebral infarction due to anterior circulation large vessel occlusion and received successful interventional recanalization.

Variables	Effective recanalization (*n* = 25)	Futile recanalization (*n* = 7)	*p* value
Age, years, (mean ± standard deviation)	62.44 ± 12.28	67 ± 11.08	0.503
Gender, Male, *n* (%)	19 (76)	6 (85.71)	0.583*
Medical history			
Hypertension, *n* (%)	13 (52)	5 (71.43)	0.360 *
Diabetes, *n* (%)	2 (8)	2 (28.57)	0.146*
Cerebral infarction, *n* (%)	4 (16)	2 (28.57)	0.451*
Coronary heart disease, *n* (%)	1 (4)	0 (0)	0.591*
Atrial fibrillation, *n* (%)	2 (8)	0 (0)	0.440*
Smoking, *n* (%)	11 (44)	2 (28.57)	0.463*
Drinking, *n* (%)	5 (20)	1 (14.29)	0.732*
mRS score before onset, mean ± standard deviation	0 (0)	0 (0)	1.000
The situation at the onset of stroke			
NIHSS score at onset, median (IQR)	4 (3–5)	5 (1–5)	0.624
Preoperative ASPECT score, median (IQR)	9 (8.5–9)	7 (6–7)	<0.001
Received intravenous thrombolysis treatment, n (%)	10 (40)	4 (57.14)	0.419*
Location of occluded blood vessels, n (%)			0.254*
M1 segment of Middle cerebral artery	13 (52)	4 (57.14)	
M2 segment of Middle cerebral artery	2 (8)	0 (0)	
Internal carotid artery	6 (24)	0 (0)	
Anterior cerebral artery	0 (0)	0 (0)	
Anterior circulation tandem lesion	4 (16)	3 (42.86)	
Collateral circulation score, median (IQR)	3 (3–4)	2 (1–2)	<0.001
eTICI grading of recanalization blood flow, *n* (%)			0.413*
2b	4 (16)	2 (28.57)	
2c	1 (4)	1 (14.29)	
3	20 (80)	4 (57.14)	
Time nodes			
Time from onset to recanalization of occluded blood vessel, min, median (IQR)	573 (323–824)	888 (437–1,476)	0.346
Time from puncture to recanalization of occluded blood vessel, min, median (IQR)	85 (67.5–118)	145 (70–165)	0.261
Pathogenesis			0.510*
Large atherosclerotic	16 (64.00)	6 (85.71)	
Cardiogenic embolism	2 (8)	0 (0)	
Other reasons	7 (28)	1 (14.29)	

^*^ Using Fisher’s exact probability method for statistics. NIHSS, National Institute of Health stroke scale; mRS, modified Rankin Scale;ASPECT, Alberta Stroke Program Early CT Score; eTICI, extended thrombolysis in cerebral infarction.

## 4 Discussion

Currently, there are few studies comparing the efficacy and safety of interventional recanalization between patients with low and non low NIHSS score in acute cerebral infarction due to anterior circulation large vessel occlusion (7). Our study found that patients with low NIHSS score acute cerebral infarction due to anterior circulation large vessel occlusion had better efficacy in interventional recanalization compared to those with non low NIHSS score, and the safety was comparable to that of patients with non low NIHSS score. The factors related to futile recanalization in patients with low NIHSS score included preoperative ASPECT score and collateral circulation score.

Unlike studies on interventional recanalization in patients with large vessel occlusion and acute cerebral infarction with a NIHSS score ≥ 6 (8–12), most current studies on interventional recanalization in patients with large vessel occlusion and acute cerebral infarction with low NIHSS score were retrospective and observational (13–16). However, international guidelines do not provide high-level recommendations for patients with acute cerebral infarction with large vessel occlusion whose NIHSS score is ≤5 points (5, 6). Exploring whether patients with acute cerebral infarction with low NIHSS score and large vessel occlusion should undergo interventional recanalization treatment, it was necessary to consider the probability of neurological deterioration in patients who had not undergone interventional recanalization treatment, whether patients can benefit from interventional recanalization treatment, the risk of complications associated with interventional recanalization and so on (16, 17).

Patients with acute cerebral infarction with large vessel occlusion and low NIHSS score may experience further exacerbation of symptoms over time (18). A study conducted by Haussen et al. (13) on patients with acute cerebral infarction with low NIHSS score and large vessel occlusion who received different treatment methods. 69% of patients received intravenous thrombolysis, 31% received interventional recanalization treatment, and 41% of patients who received intravenous thrombolysis treatment experienced neurological deterioration and needed interventional recanalization treatment. Haussen et al.’s study showed that even after receiving intravenous thrombolysis treatment, a high proportion of patients with acute cerebral infarction with large vessel occlusion with low NIHSS score would still suffer from neurological deterioration and interventional recanalization treatment was required.

Interventional recanalization therapy was a risky procedure, and data from large-scale clinical studies indicated that the main risks included cerebral hemorrhage, vascular perforation, subarachnoid hemorrhage, distal vascular embolism after reperfusion and so on. The overall incidence of complications was less than 8%, with 6% being severe complications (7–11). Compared to the expected severity of acute cerebral infarction with large vessel occlusion, this may be an acceptable incidence of complications. This suggested that if the risk of interventional recanalization was controlled within a certain range, it can benefit patients with mild stroke with large vessel occlusion. The study by Renieri et al. (14) included 134 patients with low NIHSS score who underwent interventional recanalization in acute cerebral infarction with large vessel occlusion. 73.7% of patients had successful interventional recanalization, with a symptomatic intracranial hemorrhage incidence of 5.3%. 70.9% of patients achieved neurological independence 3 months after interventional recanalization, with a mortality rate of 13.4%. The authors believed that age and successful recanalization were important predictive factors for good clinical outcomes. Our study found that the success rate of interventional recanalization in patients with low NIHSS score was 94.12%. Unlike the study by Renieri et al. (14) we only included patients with successful interventional recanalization and found that 78.13% of patients with low NIHSS score achieved neurological independence at 3 months after interventional recanalization, with a symptomatic cerebral hemorrhage incidence of 3.13%, a mortality rate of 3.13% and no patients experiencing cerebral herniation. These findings suggested that patients with low NIHSS score have a greater chance of achieving good functional prognosis once their occluded blood vessels are successfully intervened and recanalized. And we also found that there was no significant difference in the incidence of complications such as asymptomatic cerebral hemorrhage, subarachnoid hemorrhage, stroke associated pneumonia, deep vein thrombosis and gastrointestinal bleeding between the two groups of patients with low and non low NIHSS score. This indicated that the risk of interventional recanalization complications in patients with low NIHSS score was within an acceptable range. The lower preoperative ASPECT score and lower collateral circulation score were related to futile recanalization after successful interventional recanalization in patients with low NIHSS score acute cerebral infarction with anterior circulation large vessel occlusion. However, due to the small number of patients with low NIHSS score included in our study, we only conducted univariate analysis and did not conduct multivariate logistic regression analysis. As a result, we were unable to identify independent influencing factors for futile recanalization after interventional recanalization in patients with acute cerebral infarction and large vessel occlusion with low NIHSS score. Our next step should be to conduct prospective clinical studies, enrolling more patients with low NIHSS score, and randomly divide them into interventional recanalization group and drug treatment group to explore the efficacy and safety of interventional recanalization in such patients.

Previous studies had found that interventional recanalization was effective in patients with acute cerebral infarction with large vessel occlusion and low NIHSS score (19). A meta-analysis by Safouris et al. (15) included 11 eligible observational studies, including 2019 patients with acute cerebral infarction with large vessel occlusion who received interventional reperfusion therapy with a NIHSS score of ≤5, and 3,171 patients who received medication treatment. It was found that although there was an increased risk of symptomatic intracranial hemorrhage after interventional recanalization therapy, there was no difference in prognosis between the two groups of patients. The study by Abbas et al. (20) found that there was no significant difference in mRS score between the two groups of patients with acute cerebral infarction with low NIHSS score who underwent interventional recanalization treatment and conservative drug treatment at 90 days of onset. However, interventional recanalization can reduce infarct size and mortality. Toth et al. (21) completed a prospective clinical study on patients with low NIHSS score acute cerebral infarction who received interventional recanalization. A total of 20 patients were included, and the success rate of interventional recanalization was 95%. 95% of patients achieved excellent prognosis at 3 months after interventional recanalization. The authors believed that interventional recanalization was safe and feasible for patients with low NIHSS score. Our study was not entirely consistent with previous studies. In addition to finding that interventional recanalization was effective in patients with low NIHSS score acute cerebral infarction due to anterior circulation large vessel occlusion, our study also found that patients with low NIHSS score had a significantly higher 3-month postoperative good prognosis rate than those with non low NIHSS score and there was no significant difference in the complications such as symptomatic cerebral hemorrhage between the two groups of patients. This suggested that patients with low NIHSS score may have a significantly better therapeutic effect on interventional recanalization than those with non low NIHSS score. This is a unique finding of our study. Our study supported that patients with acute cerebral infarction due to large vessel occlusion with low NIHSS score could have the opportunity to benefit from interventional recanalization if there is an opportunity to benefit from interventional recanalization after detailed evaluation. However, using a NIHSS score of ≤5 as the standard for low NIHSS score may not accurately reflect the volume of the patient’s infarction, which means that a low NIHSS score does not necessarily mean a small infarction volume. The NIHSS score can only reflect the severity of a patient’s symptoms. In practical work, we also need to pay attention to the imaging characteristics of the patient’s infarction volume and occluded blood vessels, speculate on the most likely pathogenesis, and predict the possibility of worsening of patients’ symptoms. After analyzing multiple factors and considering the potential benefits and risks of the patient receiving interventional recanalization treatment, it is ultimately determined whether the patient should receive interventional recanalization treatment. This also means that establishing a predictive model for the aggravation of symptoms in patients with acute cerebral infarction and large vessel occlusion with low NIHSS score is also an important research direction in the next step.

There are some limitations in this study. Firstly, this study was a single center, retrospective study. Secondly, the number of low NIHSS score cases included in this study was limited, and only patients who received successful recanalization were included, while patients who received failed interventional recanalization were not included, making it difficult to avoid selection bias. Thirdly, there may be some changes including potential advancements in treatment protocols, advances in treatment equipment, patient demographics and other external factors that may have occurred during the study period that could have influenced the results. It is necessary to conduct a prospective, randomized controlled clinical study on whether patients with low NIHSS score acute cerebral infarction due to anterior circulation large vessel occlusion should receive interventional recanalization treatment.

## 5 Conclusion

The successful interventional recanalization for patients with low NIHSS score acute cerebral infarction due to anterior circulation large vessel occlusion had good therapeutic effect, and the safety was comparable to that of non low NIHSS score patients. The factors that related to futile recanalization in patients with low NIHSS score included preoperative ASPECT score and collateral circulation score.

## Data Availability

The original contributions presented in the study are included in the article/supplementary material, further inquiries can be directed to the corresponding author.
